# Assessment of Mental Health and Quality of Life Status of Undergraduate Students in Indonesia during COVID-19 Outbreak: A Cross-Sectional Study

**DOI:** 10.3390/ijerph191912011

**Published:** 2022-09-22

**Authors:** Hidayah Karuniawati, Nila Sari, Md. Sanower Hossain, Wan Ismahanisa Ismail, Aniq Hudiyah Bil Haq, Tri Yulianti, Taufik Taufik, Gardhika Rizky Sudarsono

**Affiliations:** 1 Department of Pharmacology and Clinical Pharmacy, Faculty of Pharmacy, Universitas Muhammadiyah Surakarta, Surakarta 57102, Indonesia; 2 Centre for Sustainability of Ecosystem and Earth Resources (Pusat ALAM), Universiti Malaysia Pahang, Kuantan 26300, Malaysia; 3 Faculty of Health Science, Universiti Teknology MARA, Cawangan Pulau Pinang, Kampus Bertam, Kepala Batas 13200, Malaysia; 4 Faculty of Psychology, Universitas Muhammadiyah Kalimantan Timur, Samarinda 75124, Indonesia; 5 Faculty of Psychology, Universitas Muhammadiyah Surakarta, Surakarta 57102, Indonesia

**Keywords:** DASS-21, environmental health, mental health, psychological health, quality of life, WHOQOL-BREF

## Abstract

The COVID-19 pandemic globally impacted physical, spiritual, and mental health (MH). The consequences significantly affected students’ quality of life (QoL) too. This cross-sectional study assessed MH status and its relationship to the QoL of college students in Indonesia. This study collected data (September 2021–April 2022) online using the depression, anxiety, and stress scale-21 (DASS-21) to measure MH and the world health organization quality-of-life scale (WHOQoL-BREF) to measure the QoL. The data were analysed using SPSS with a bivariate and multivariate linear regression test. A total of 606 respondents participated in this study, with the majority being women (81.0%), aged 21–27 years (44.3%), and unmarried (98.5%) respondents. We observed 24.4% (n = 148) moderate depression, 18.3% (n = 111) very severe anxiety, and 21.1% (n = 128) moderate stress status. The QoL measurement determined that a moderate QoL in the physical and environmental health domains (>70%) and poor QoL in the psychological health domain (58.3%) were found. Gender, age, family support, history of COVID-19 diagnosis, family with COVID-19 diagnosis, vaccination status, and physical symptoms are significantly associated with MH status and QoL (*p*-value < 0.05). This study demonstrated that COVID-19 was negatively related to college students’ MH and QoL. Targeted interventions may be needed to ameliorate both MH and QoL.

## 1. Introduction

Since the announcement of the outbreak of COVID-19 worldwide caused by a virus known as severe acute respiratory syndrome coronavirus 2 (SARS-CoV-2) by the World Health Organization (WHO) on 12 March 2020, Indonesia has become one of the countries that have been severely affected by COVID-19 and it has impacted on physical, spiritual, and mental health (MH) [1]. In the two years since the beginning of the pandemic, the number of positive cases of COVID-19 in Indonesia has reached 6,216,621 million, with a death toll of 157,028 (as of 7 August 2022) [2,3]. With the rapid spread of COVID-19 and the increasing number of cases from day to day globally, the Government of Indonesia has made policies to limit population movement and social activities to reduce the spread of the virus. One of the steps taken by the Indonesian government to prevent the spread of the virus was to close academic institutes, ranging from basic to higher education [4]. According to Zhang et al. (2020), the inhibition of direct communication (face to face) will cause a social psychological impact on society. Therefore, the closure of the academic institutes certainly caused anxiety among the students; subsequently, their MH and QoL were affected [5].

The pandemic condition that causes students to be isolated at home and unable to do activities outside the house causes them to feel afraid of being exposed to the virus, and the lack of knowledge about the SARS-CoV-2 can cause mental disorders for these students [6]. At the beginning of the lockdown, Wang et al. (2020) mentioned that students in China are at greater risk of stress, anxiety, and depression due to the COVID-19 pandemic, compared to older people [7]. Wathelet et al. (2020) also mentioned the high incidence of mental disorders among students, especially those undergoing quarantine [8]. During this pandemic, sleep disturbances and MH deterioration occurred significantly among health students in Greece [9] and many more countries [10,11,12].

In addition to MH, QoL is also something that needs to be considered during a pandemic. The COVID-19 pandemic has substantially impacted people’s QoL, as well as their physical and MH [13]. Prolonged anxiety due to uncertainty about when the pandemic will end and changes in daily routine activities cause changes in the community’s QoL, especially students (young adults) [14]. The results of existing research in Malaysia and Poland showed that during the pandemic, the QoL of students was low, with various influencing factors, such as frustration because their learning was disrupted. Living in areas with a high prevalence of COVID-19 had a high rate of depression and stress [11,14].

Previous studies revealed that sociodemographic characteristics, especially age and gender, were significantly associated with students’ MH and quality of life [10,15,16]. Physical symptoms, including fever, cough, and myalgia, were significantly associated with depression, anxiety, and stress (*p* < 0.05) [16]. In addition, there were many factors, such as age, gender, marital status, education, occupation, income, residential area, close contact with people with COVID-19, comorbid physical and MH problems, exposure to COVID-19-related news and social media, coping styles, stigma, psychosocial support, health communication, confidence in health services, personal protective measures, risk of contracting COVID-19 and perceived likelihood of survival associated with MH problems [17]. During the pandemic, family and social support were also related to MH issues [11]. This research hypothesizes that sociodemographic characteristics, physical symptoms, and other factors, such as family support, history, and family with COVID-19 diagnosis; comorbid disease; and vaccination status, are related to MH and QoL of students in Indonesia.

Research on measuring the MH and QoL during the pandemic, especially for students, has not been extensively conducted, particularly in Indonesia. Even though students are part of the community and relatively significantly affected, their MH and QoL evaluation was severely neglected. Therefore, this study was designed to assess the MH and QoL of students in Indonesia during the COVID-19 pandemic. Since such studies have not been widely carried out in Indonesia, the results of this study can be used to assess MH, QoL, and the factors that influence them. Furthermore, knowing the degree of MH and QoL, as well as the factors related to MH and QoL, can be used as a reference in conducting interventions in the form of prevention and treatment of mental disorders, which are expected to improve students’ QoL.

## 2. Materials and Methods

### 2.1. Study Site

Indonesia is the 14th largest country and the largest archipelagic country in the world, with an area of 1,904,569 km^2^, and the 6th largest island country, with 17,504 islands. Indonesia is also the 4th most populous country in the world, with a population of 270,203,917 in 2020 [18]. Indonesia is bordered by several neighbouring countries in Southeast Asia, the Australian continent, and Oceania. Indonesia borders on land with Malaysia on the islands of Borneo and Sebatik, Papua New Guinea on the island of Papua, and Timor Leste on the island of Timor. Indonesia has the five largest islands, including Java, Kalimantan, Sumatra, Sulawesi, and Irian Jaya. Indonesia is an agricultural country where the livelihood of the majority of the population is farming [18,19].

Indonesia is one of the largest populated countries affected by the COVID-19 pandemic, in all aspects of life, including national health status and slowing economic growth. In Indonesia, cases have increased since June 2021, with the highest peak in July 2021, with 49,509 new cases and 1893 deaths, with an average of 1646 every seven days [3]. Then, it decreased from October 2021 to January 2022. However, cases increased again in January to reach the highest number of new cases in February 2022 with 59,384 new cases at an average of 7 days, as many as 55,110 new cases, and the highest death in March 2022 with the number of deaths of 401 cases with an average of 310 cases of death per week [3].

### 2.2. Study Design, Data Collection, and Sample Size

This research is a cross-sectional study with a purposive sampling technique. Data collection was done online using a validated questionnaire consisting of the DASS-21 and WHOQoL-BREF questionnaires [20,21]. The DASS-21 questionnaire was translated using the forward-backward translation method. It was translated from English to Indonesian by an English–Indonesian sworn translator and then re-translated into English by another certified English–Indonesian translator to ensure validity and accuracy [22]. Furthermore, the translation process was not carried out on the WHOQoL-BREF questionnaire because WHO provided it in Indonesian [23]. The questionnaire was presented in the form of a google form in the Indonesian language and then distributed online via social networking apps, such as WhatsApp, Instagram, and Line. Data were collected from September 2021 and April 2022. The inclusion criteria in this study were active undergraduate students aged ≥17 years who could fill out a questionnaire via a google form and were willing to participate. Several efforts were made to get the appropriate respondents. Inclusion and exclusion criteria for respondents were written at the beginning of the questionnaire, and information about rewards for lucky participants was also stated. In addition to sharing the questionnaire link on social media groups consisting of students, group members were also asked to share the link with other friends. Involving students in the research team helps maximize the recruitment of respondents. Raosoft’s sample size calculator was used to determine the sample size for this study [21,24]. For more than 270 million people in Indonesia, the minimum estimated sample size was 385.

### 2.3. Research Instrument

The instrument used in this study was a questionnaire consisting of 3 parts. The first part contains demographic data, the second deals with questions measuring students’ QoL, and the third part consists of questions that measure students’ MH status—measuring students’ mental status using the DASS 21 questionnaire. Measurement of MH with DASS 21 is divided into three domains. Each domain has seven questions, namely depression (Q3, Q5, Q10, Q13, Q16, Q17, Q21), anxiety (Q2, Q4, Q7, Q9, Q15, Q19, Q20), and stress (Q1, Q6, Q8, Q11, Q12, Q14, Q18). The dysphoria, hopelessness, devaluation of life, self-deprecation, lack of interest or involvement, anhedonia, and inertia are all evaluated by the depression scale. The anxiety scale measures autonomic arousal, skeletal muscle effects, situational anxiety, and subjective sensation of anxious affect. The persistent nonspecific arousal levels are sensitive to the stress scale. It evaluates tension, anxiety, and the propensity to become easily irritated, irritable, overreactive, and impatient. The responses to this questionnaire are recorded on a Likert scale from 0 (did not apply at all) to 3 applied very much or most of the time). To determine the final score, multiply the DASS-21 score by two. After that, the interpretation of the results of each domain is described in five categories, namely the normal, mild, moderate, severe, and very severe. Depression was categorized as normal (0–9), mild (10–13), moderate (14–20), severe (21–27), and very severe (≥28). As for anxiety, the categories are normal (0–7), mild (8–9), moderate (10–14), severe (15–19), and very severe (≥20). In the stress domain, the categories are normal (0–14), mild (15–18), moderate (19–25), severe (26–33), and very severe (≥34) [20,21].

The WHOQoL-BREF questionnaire used to assess the quality of life is already available in Indonesian, so there is no need for further translation [23]. Responses to the quality-of-life questionnaire are 1–5, with questions number 3, 4, and 26 being unfavourable questions with a score of 5 to 1. Measuring QoL is divided into four domains, namely physical health (Q3, Q4, Q10, Q15, Q16, Q17, Q18), psychological (Q5, Q6, Q7, Q11, Q19, Q26), social relationship (Q20, Q21, Q22), and environment (Q8, Q9, Q12, Q13, Q14, Q23, Q24, Q25) with raw minimum and the maximum score for each domain is 7–35, 6–30, 3–15, and 8–40, respectively. After the scores in each domain are added up and then transformed to a score of 0–100 (0 = the worst score, 100 = the best score), with a formula as in Equation (1). Higher scores indicate a higher QoL on a scale of one to four for each domain [21,24]. For example, a score of less than 45 categorizes poor, 45–65 is moderate, and more than 65 is good [25].

(1) $$Total\;score\;\left(\%\right)=\frac{Obtained\;score-least\;possible\;score}{Maximum\;score-Least\;possible\;score}\times 100$$

The questionnaire’s validity and reliability were evaluated on 30 respondents. The Pearson bivariate method was used to assess the data validity, while Cronbach’s alpha was used to assess the data reliability. Results obtained a value of sig two-tailed 0.05 or R count >0.361 from the DASS 21 questionnaire, indicating that the questionnaire is valid, and a score of 0.918 for Cronbach Alpha, indicating that the DASS 21 questionnaire is reliable. In addition, the results of the validity and reliability test of the WHOQoL-BREF questionnaire are also valid and reliable. An R count is more than the R table with an R count range of 0.425–0.749. At the same time, Cronbach’s Alpha test result is 0.931 [26,27].

### 2.4. Ethical Clearance

The Medical and Health Research Ethics Committee of the Faculty of Medicine at Universitas Muhammadiyah Surakarta approved the study protocol before the study’s execution (Reference No. 3725/B.1/KEPK-FKUMS/IX/2021). The nature of the study was explained to the respondents in writing, who were asked to sign an informed consent form by clicking “agree to participation” to confirm their participation.

### 2.5. Data Analysis

Online-based questionnaires were collected, and data were analysed using the Statistical Package for the Social Sciences (SPSS) version 25 (International Business Machines Corporation, New York, NY, USA). Descriptive statistics are used to analyse respondents’ demographic data. First, the normality of the data was tested with Kolmogorov Smirnov. A bivariate correlation test (Mann–Whitney and Kruskal–Wallis) determined the relationship between demographic characteristics with MH status and the QoL of respondents. Mann–Whitney determined the relationship of the two groups’ independent variables (e.g., gender, marital status, faculty, and perceived physical symptoms) to the dependent variable. At the same time, Kruskal–Wallis was used to evaluate the relationship between independent variables of more than two groups, such as age and the dependent variable. Variable results of the bivariate analysis with a *p*-value <0.25 were followed by a multivariate analysis tests with a linear regression method [28]. The correlation between MH and QoL was tested with the Spearman test correlation and Bonferroni correction. A statistically significant difference between groups was determined at the 95% confidence level (*p*-value < 0.05).

## 3. Results

The number of respondents who were willing to fill out the questionnaire in this study was 606, with the majority being female respondents amounting to 81.0% (n = 491) respondents, 44.3% (n = 268) 21–27 years old, and 98.5% unmarried. The demographic data of the respondents are presented in Table 1.

Based on Table 2 and Table 3, although the majority of respondents (42.1%) were categorized as normal depression with a score between 0 and 9, almost a quarter of respondents experienced moderate depression (24.4%) with a score of 14–20 and 6.8% experienced a very severe depression with score 28–42. More than half of the respondents sometimes experienced that they could not seem to experience any positive feeling at all, had difficulty working up the initiative to do things, felt downhearted and blue, or were unable to become enthusiastic about anything. More than 10% of respondents often experienced difficulty working up the initiative to do things, felt that they had nothing to look forward to, felt downhearted and blue, or were unable to become enthusiastic about anything. For the anxiety domain, 34.3% (n = 208) of participants reported moderate depression with a score of 10–14, and almost a fifth of respondents reported a very severe level of anxiety with a score of 20–42. More than a third of respondents experienced dryness of their mouth, trembling, worry about situations, panic, a sense of heart rate increase, and feeling scared without any good reason. The stress domain result revealed that most respondents experienced mild stress (40.8%), and more than a fifth suffered moderate stress (21.1%). About 20% of respondents often experienced difficulty winding down, tended to overreact to situations, used much nervous energy, got agitated, and were intolerant of anything.

The QoL of more than 70% of respondents was at a moderate level in the domain of physical health and environmental health. A total of 35.6% of respondents said they do not have enough energy to carry out daily activities. More than 30% of respondents also stated that they feel insecure in their daily lives, feel that the physical environment is unhealthy, do not have enough money to meet their needs, lack opportunities in recreational activities, and lack availability of required information. Although 42.4% of respondents’ QoL in the social relationship domain were in a moderate category, more than half of the respondents (58.3%; n = 353) stated that they were poor in the psychological health domain. Respondents stated that they do not enjoy life, feel that life is less meaningful, have difficulty concentrating, and are not satisfied with their bodily appearance (Table 4 and Table 5).

In general, the levels of depression, anxiety, and stress in women are higher, and their QoL is lower than in men (Table 6). Faculty and marital status are unrelated to students’ mental status and QoL. The variables that significantly affect students’ mental status (depression, anxiety, and stress) are gender, family support, history of COVID-19 diagnosis, and physical symptoms, such as headache, myalgia, and sore throat. Age, family support, family with COVID-19 diagnosis, vaccination status, and experience of suffering physical symptoms, such as sore throat and cough, influenced the QoL significantly (Table 7 and Table 8).

A spearman test was conducted to determine the correlation between MH status and students’ QoL. Based on the results of the spearman test for the correlation between MH status and QoL, it was found that MH status was significantly correlated with the QoL in the domains of physical health, psychology, and social relationships (*p* < 0.05). In contrast, MH was not significantly correlated to the QoL in the environmental health domain (*p* > 0.05) (Table 9).

## 4. Discussion

This study aims to identify and determine the relationship between MH status and the QoL of students in Indonesia during the COVID-19 pandemic and its associated factors. This study found that the majority of the respondents (42.1%) suffered normal levels of depression, which is a good sign of community MH. For the anxiety domain, almost one-fifth of respondents reported a very severe level of anxiety, which is alarming. The stress domain result revealed that more than one-fifth suffered moderate stress (21.1%). In general, depression, anxiety, and stress levels in women are higher than in men. Compared to men, women’s QoL is lower. This is in line with a study conducted in the United Kingdom and Saudi Arabia, where there were high levels of anxiety and depression in university students, with more than 50% experiencing levels above the clinical cut-offs and females scoring significantly higher than males [10,29]. Several other studies conducted in Pakistan, China, Hungary, the United States, and Indonesia also reported a significant impact on university students’ MH due to the COVID-19 outbreak. College students reported feeling more anxious, tired, and depressed than before the pandemic [12,16,30,31,32,33].

This study also revealed that most college students report a moderate QoL in the domain of physical health and environmental health. The existence of social distancing implemented to prevent the spread of the COVID-19 virus causes limitations in physical and social activities, including leisure activities and the sufficiency of the family’s financial needs. This is consistent with a study conducted in the UK, which stated that low resilience was associated with restriction and isolation, reducing the chances of engaging in beneficial coping strategies and activities rather than enduring personality characteristics. Higher levels of distress are associated with lower levels of exercise, higher rates of tobacco use, and several life events associated with the pandemic and lockdown, such as cancelled events, worsening personal relationships, and financial problems [14]. Furthermore, the lockdown and university closures have forced students to study at home. In a study conducted in Indonesia, 34.38% of students felt depressed while studying at home. Other emotions were anger (0.39%), surprise (7.91%), and fear (15.81%) [30].

The QoL of more than 50% of respondents in the psychological health domain is in the poor category, where pleasure in life, the meaning of life, concentration, and self-acceptance begin to decrease. A qualitative study in the USA showed that out of 195 students, 138 (71%) showed increased stress and anxiety due to the COVID-19 outbreak. In addition, several stressors were identified that contributed to increased stress, anxiety, and depressive thoughts among students. These included fears and concerns about their health and those of their loved ones (91% reported a negative impact of the pandemic), difficulty concentrating (89%), disturbed sleep patterns (86%), decreased social interaction due to physical distance (86 %), and increased concern about academic performance (82%) [33].

The study results show that MH status is related to students’ QoL. Student MH status scores significantly negatively correlate with each dimension of QoL, as measured using WHOQOL-BREF. Depression has a negative and significant correlation with physical health domains r = −0.393 (*p* < 0.001), psychology r = −0.161 (*p* < 0.001), and social relations r = −0.400 (*p* < 0.001) but has no significant impact on environmental health r = −0.040 (*p* = 0.325). This means a significant relationship exists between depression and students’ physical, psychological, and social health. The higher the student’s depression score, the lower the student’s QoL [11]. These results are consistent with a study conducted in Macau, Hong Kong, and mainland China which stated that, compared to the “No depression” group, students with depression had significantly lower QoL scores in the physical, psychological, social, and environmental domains [34]. Depressed people tend to isolate themselves from their surroundings. They get tired quickly, struggle to go asleep, have trouble staying awake, experience uncontrollable and unpleasant emotions, and lack interest in learning in students due to impaired concentration [35]. For students with low levels of depression, the strategy used is to divide time between studying and exercising, even though most activities are carried out at home, in line with research conducted by Abdullah et al. [11]. During the lockdown in Malaysia, it was found that there were changes in the daily lives of students in terms of activities that affect physical activities, such as exercising with family, which became a new routine for students to overcome boredom and maintain their physical health during the pandemic [11].

Anxiety and stress are also significantly negatively correlated with physical, psychological, and social domains. This indicates that the higher the level of anxiety and stress of students, the lower their QoL, especially in the physical, psychological, and social domains. Stress is a condition that often occurs in everyday life, especially with the pandemic as a stressor that increasingly triggers the emergence of psychological pressures. The forms of stress experienced by students during the pandemic are difficulty concentrating because they have to be isolated at home, difficulty studying lecture material, and worries about their future [36]. Another study in a public university in North Carolina showed that the problem with distanced learning and social isolation contributed to the increases in depression and anxiety [37].

In this study, MH and QoL were influenced by age, gender, family support, history of COVID-19 diagnosis, family with COVID-19 diagnosis, vaccination status, and physical symptoms, such as headache, myalgia, sore throat, and cough. A study in the United States revealed that being a woman and knowing someone infected with COVID-19 predicted higher levels of psychological impact among university students [38]. The possible symptoms of COVID-19 infection include fever or chills, cough, shortness of breath, muscle or body aches, headache, loss of taste or smell, diarrhoea, and sore throat [39,40]. The COVID-19 symptoms may persist and continue for weeks or months [41,42]. A multicentre prospective cohort study reported that the burden of persistent symptoms was strongly correlated with poorer long-term health status, lower QoL, and psychological distress in patients with moderate acute COVID-19 [43]. Support from family, neighbours, and colleagues plays an important role in helping strengthen people or families who are positive for COVID-19 in living their daily lives. The support is in the form of moral and material support, including emotional support (asking for news, encouraging), instrumental support (meeting basic needs, household needs, and medicine), information support, and logistical and financial assistance [44].

Universities, the institutions closest to and associated with students, can take roles related to MH and improving students’ QoL, especially during the COVID-19 outbreak. Universities can carry out several strategies for promotion and prevention and the therapeutic process related to students’ MH disorders. Psychoeducation is one of the promotions of individual action for good MH, which can be broken down into recommendations for general well-being, dealing with stress and crises, providing tips for healthy lifestyles, explaining general emotional reactions to epidemics, advising on how to cope with isolation and quarantine periods, and describing warning signs that require personal assessment or even emergency treatment [45]. Screening can also be done, especially in high-risk populations, for example having a history of mental disorders, students with poor economic conditions, and students with divorced parents. Screening should include symptoms of depression, anxiety, stress, suicidal ideation and behaviour, and insomnia, which can be early markers of mental disorders. Counselling is carried out with the aim of providing psychological support and even clinical psychology and psychiatric care. Referral assistance and funding can be applied if students require continued therapy and medication [45,46].

Teaching-related changes emphasizing MH can also be applied by implementing a fun online learning process. Features described for this learning environment include active, interactive learning, with discussion panels and group work, and inclusive learning, in the sense that the student leads and participates in teaching-related decisions [46]. Explicit instruction about academic activities can reduce uncertainty and anxiety and help students manage their time. Remedial programs and temporary suspension of payments can be included to offset possible disruptions caused by the pandemic [47,48]. Furthermore, students are encouraged to apply good time management to help balance study, rest, exercise, fun, and leisure activities [46].

## 5. Strengths and Limitations of Study

This study has received responses more than 57% higher than the minimum sample size (n = 385). The data were obtained from eighteen different provinces. Since this study collected data by spreading online survey forms using social networking sites, the actual distribution and response rate were not possible to calculate. There is the possibility of sample bias due to the distribution of the survey questionnaire online, as the respondents cannot be described, and biased respondents may re-enrol themselves in the sample.

This study examined psychiatric symptoms using a self-reported questionnaire and did not make a clinical diagnosis. The DASS 21 and WHO QoL questionnaires are initial screening and require further examination by an MH professional to determine a more accurate diagnosis. Despite a few limitations, our study is the first one that has laid a foundation to understand better the students’ MH, QoL, the correlation between MH and QoL, and its associated factors. These findings could guide developing policies to address psychological problems brought on by the COVID-19 pandemic. Our results may also be used to create effective psychological and non-psychological interventions to overcome students’ MH problems and minimize the negative impact on quality of life.

## 6. Conclusions

This study revealed that students reported normal to very severe levels of depression, anxiety, stress, and good-to-poor QoL during the COVID-19 outbreak. Furthermore, gender, age, family support, history of COVID-19 diagnosis, family with COVID-19 diagnosis, vaccination status, and physical symptoms, such as headache, myalgia, sore throat, and cough, are significantly associated with MH and QoL status. The findings of this study are important for improving our understanding of the MH status and QoL of university students.

The level of MH correlates to the QoL status. Urgent efforts by health officials are needed to implement some strategies that may include physical, psychological, and drug treatment to address MH issues among college students and improve their QoL. Here, we suggest some interventions that could be immediately implemented nationwide by the government and universities: (i) implementing a teaching-related process that emphasizes students’ MH; (ii) providing explicit instruction about academic activities, remedial programs, and suspension of payments or financial support for students; (iii) promoting and preventing mental disorders through psychoeducation; (iv) screening for early detection; (v) since being a women, being aged 18–20 years old, having a lack of family support, having a history of COVID-19 diagnosis, and having physical symptoms, such as headache, myalgia, sore throat, and cough, are related for the possibility of having a mental disorder, the promotion and prevention programs might be a prioritized for these groups; (vi) for students who should use medicines to treat mental disorders, counselling to medication-related adherence and management of medication side effects if they arise is essential so that therapeutic goals can be achieved optimally; (vii) it is important to monitor the MH of students during and after the outbreak and evaluate the success of programs.

## Figures and Tables

**Table 1:** Demographic characteristics of respondents (n = 606).

Variables	Number (n = 606)	Percentage (%)
**Gender**		
Man	115	19.0
Women	491	81.0
**Age**		
<18	5	0.8
18–20	333	54.9
21–27	268	44.3
**Marital Status**		
Married	9	1.5
Single **Name of Faculty** Health Non-Health **Domicile (in Province)** Central Java	597 182 424 331	98.5 30.0 70.0 54.6
East Kalimantan East Java West Java Jakarta Yogyakarta Central Kalimantan West Kalimantan Lampung Bali South Sumatera Papua Jambi South Kalimantan Nort Kalimantan West Nusa Tenggara Riau West Sumatera	138 72 18 14 8 8 3 3 2 2 1 1 1 1 1 1 1	22.8 11.9 3.0 2.3 1.3 1.3 0.5 0.5 0.3 0.3 0.2 0.2 0.2 0.2 0.2 0.2 0.2

**Table 2:** The score and level of college students’ MH status (n = 606).

Variable	Level of MH Status									
	Normal N (%)	Score	Mild N (%)	Score	Moderate N (%)	Score	Severe N (%)	Score	Very Severe N (%)	Score
Depression	255 (42.1)	0–9	112 (18.5)	10–13	148 (24.4)	14–20	50 (8.3)	21–27	41 (6.8)	28–42
Anxiety	149 (24.6)	0–7	54 (8.9)	8–9	208 (34.3)	10–14	84 (13.9)	15–19	111 (18.3)	20–42
Stress	163 (26.9)	0–14	247 (40.8)	15–18	128 (21.1)	15–19	50 (8.3)	26–33	18 (3.0)	34–42

**Table 3:** Responses to the questionnaire on MH status (n = 606).

Domain	Questions	Respondent’s Answer				Median
		0 (%)	1 (%)	2 (%)	3 (%)	
Depression	(Q3) I couldn’t seem to experience any positive feeling at all	230 (38.0)	305 (50.3)	57 (9.4)	14 (2.3)	1.0
	(Q5) I found it difficult to work up the initiative to do things	137 (22.6)	336 (55.4)	108 (17.8)	25 (4.1)	1.0
	(Q10) felt that I had nothing to look forward to	224 (37.0)	268 (44.2)	83 (13.7)	31 (5.1)	1.0
	(Q13) I felt down-hearted and blue	160 (26.4)	318 (52.5)	91 (15.0)	37 (6.1)	1.0
	(Q16) I was unable to become enthusiastic about anything	204 (33.7)	306 (50.5)	66 (10.9)	30 (5.0)	1.0
	(Q17) I felt I wasn’t worth much as a person	301 (49.7)	211 (34.8)	55 (9.1)	39 (6.4)	1.0
	(Q21) I felt that life was meaningless	339 (55.9)	178 (29.4)	52 (8.6)	37 (6.1)	0.0
Anxiety	(Q2) I was aware of dryness of my mouth	204 (33.7)	272 (44.9)	102 (16.8)	28 (4.6)	1.0
	(Q4) I experienced breathing difficulty (e.g., excessively rapid breathing, breathlessness in the absence of physical exertion)	414 (68.3)	147 (24.3)	36 (5.9)	9 (1.5)	0.0
	(Q7) I experienced trembling (e.g., in the hands)	258 (42.6)	224 (37.0)	89 (14.7)	35 (5.8)	1.0
	(Q9) I was worried about situations in which I might panic and make a fool of myself	112 (18.5)	249 (41.1)	170 (28.1)	75 (12.4)	1.0
	(Q15) I felt I was close to panic	123 (20.3)	300 (49.5)	130 (21.5)	53 (8.7)	1.0
	(Q19) I was aware of the action of my heart in the absence of physical exertion (e.g., sense of heart rate increase, heart missing a beat)	316 (52.1)	204 (33.7)	66 (10.9)	20 (3.3)	0.0
	(Q20) I felt scared without any good reason	209 (34.5)	234 (38.6)	111 (18.3)	52 (8.6)	1.0
Stress	(Q1) I found it hard to wind down	107 (17.7)	351 (57.9)	122 (20.1)	26 (4.3)	1.0
	(Q6) I tended to over-react to situations	129 (21.3)	313 (51.7)	125 (20.6)	39 (6.4)	1.0
	(Q8) I felt that I was using a lot of nervous energy	150 (24.8)	282 (46.5)	121 (20.0)	53 (8.7)	1.0
	(Q11) I found myself getting agitated	112 (18.5)	327 (54.0)	121 (20.0)	46 (7.6)	1.0
	(Q12) I found it difficult to relax	131 (21.6)	327 (54.0)	108 (17.8)	40 (6.6)	1.0
	(Q14) I was intolerant of anything that kept me from getting on with what I was doing	101 (16.7)	285 (47.0)	163 (26.9)	57 (9.4)	1.0
	(Q18) I felt that I was rather touchy	137 (22.6)	297 (49.0)	117 (19.3)	55 (9.1)	1.0

0 = did not apply to me at all, 1 = applied to me to some degree or some of the time, 2 = applied to me a considerable degree or a good part of the time, 3 = applied to me very much or most of the time.

**Table 4:** The number of questions and level of college students’ QoL status (n = 606).

Domain	Number of Questions	Level		
		Poor (<45) n (%)	Moderate (45–65) n (%)	Good (>65) n (%)
Physical health	7	36 (5.9)	432 (71.3)	138 (22.8)
Psychological health	6	353 (58.3)	226 (37.3)	27 (4.5)
Social relationship	3	83 (13.7)	257 (42.4)	266 (43.9)
Environmental Health	8	144 (23.8)	428 (70.6)	33 (5.4)

**Table 5:** Responses to the questionnaire on quality-of-life status (n = 606).

Domain	Question	Respondent’s Answer (%)					Median
		1	2	3	4	5	
Physical Health	(Q3) To what extent do you feel that physical pain prevents you from doing what you need to do?	5 (0.8)	18 (3.0)	119 (19.6)	393 (64.9)	71 (11.7)	4.0
	(Q4) How much do you need any medical treatment to function in your life?	4 (0.7)	8 (1.3)	36 (5.9)	235 (38.8)	323 (53.3)	5.0
	(Q10) Do you have enough energy for everyday life?	85 (14.0)	216 (35.6)	267 (44.1)	37 (6.1)	1 (0.2)	3.0
	(Q15) How well are you able to get around?	9 (1.5)	40 (6.6)	474 (78.2)	83 (13.7)	0 (0)	3.0
	(Q16) How satisfied are you with your sleep?	23 (3.8)	116 (19.1)	215 (35.5)	187 (30.9)	65 (10.7)	3.0
	(Q17) How satisfied are you with your ability to perform your daily living activities?	11 (1.8)	51 (8.4)	299 (49.3)	213 (35.1)	32 (5.3)	3.0
	(Q18) How satisfied are you with your capacity for work?	10 (1.7)	58 (9.6)	267 (44.1)	214 (35.3)	57 (9.4)	3.0
Psychological	(Q5) How much do you enjoy life?	121 (20.0)	215 (35.5)	232 (38.3)	37 (6.1)	1 (0.2)	2.0
	(Q6) To what extent do you feel your life to be meaningful?	134 (22.1)	244 (40.3)	187 (30.9)	40 (6.6)	1 (0.2)	2.0
	(Q7) How well are you able to concentrate?	29 (4.8)	242 (39.9)	298 (49.2)	37 (6.1)	0 (0)	3.0
	(Q11) Are you able to accept your bodily appearance?	122 (20.1)	237 (39.1)	201 (33.2)	46 (7.6)	0 (0)	2.0
	(Q19) How satisfied are you with yourself?	14 (2.3)	60 (9.9)	191 (31.5)	241 (39.8)	100 (16.5)	4.0
	(Q26) How often do you have negative feelings, such as blue mood, despair, anxiety, depression?	18 (3.0)	126 (20.8)	224 (37.0)	211 (34.8)	27 (4.5)	3.0
Social Relationship	(Q20) How satisfied are you with your personal relationships?	16 (2.6)	70 (11.6)	227 (37.5)	251 (41.4)	42 (6.9)	3.0
	(Q21) How satisfied are you with your sex life?	9 (1.5)	39 (6.4)	374 (61.7)	137 (22.6)	47 (7.8)	3.0
	(Q22) How satisfied are you with the support you get from your friends?	5 (0.8)	37 (6.1)	227 (37.5)	248 (40.9)	89 (14.7)	4.0
Environment	(Q8) How safe do you feel in your daily life?	68 (11.2)	230 (38.0)	262 (43.2)	45 (7.4)	1 (0.2)	3.0
	(Q9) How healthy is your physical environment?	92 (15.2)	219 (36.1)	239 (39.4)	55 (9.1)	1 (0.2)	2.0
	(Q12) Have you enough money to meet your needs?	66 (10.9)	203 (33.5)	306 (50.5)	31 (5.1)	0 (0)	3.0
	(Q13) How available to you is the information that you need in your day-to-day life?	82 (13.5)	256 (42.2)	240 (39.6)	27 (4.5)	1 (0.2)	2.0
	(Q14) To what extent do you have the opportunity for leisure activities?	40 (6.6)	306 (50.5)	233 (38.4)	27 (4.5)	0 (0)	2.0
	(Q23) How satisfied are you with the conditions of your living place?	4 (0.7)	28 (4.6)	174 (28.7)	290 (47.9)	110 (18.2)	4.0
	(Q24) How satisfied are you with your access to health services?	5 (0.8)	26 (4.3)	229 (37.8)	285 (47.0)	61 (10.1)	4.0
	(Q25) How satisfied are you with your mode of transportation?	2 (0.3)	32 (5.3)	207 (34.2)	287 (47.4)	78 (12.9)	4.0

**Table 6:** Association of demographic characteristics and depression, anxiety, stress, physical health, psychological health, social relations, and environment.

		DASS-21						WHOQOL-BREF							
Characteristic	n	Depression		Anxiety		Stress		Physical Health		Psychological Health		Social Relations		Environment	
		Mean ± SD	*p*-Value	Mean ± SD	*p*-Value	Mean ± SD	*p*-Value	Mean (±SD)	*p*-Value	Mean ± SD	*p*-Value	Mean ± SD	*p*-Value	Mean ± SD	*p*-Value
Gender *															
Man	115	9.76 ± 7.54	0.008	9.44 ± 6.38	<0.001	12.83 ± 6.88	<0.001	61.24 ± 9.78	0.024	43.05 ± 11.97	0.397	63.19 ± 17.66	0.100	48.78 ± 8.37	0.971
Woman	491	12.35 ± 9.06		13.50 ± 8.09		16.87 ± 8.90		59.46 ± 9.58		41.99 ± 11.14		60.27 ± 15.43		49.18 ± 9.39	
Age **															
<18	5	11.60 ± 6.84	0.074	11.20 ± 4.15	0.176	16.80 ± 3.90	0.105	54.98 ± 4.78	0.082	40.09 ± 9.59	0.009	63.34 ± 20.04	0.929	44.52 ± 16.37	0.036
18–20	333	12.39 ± 8.50		13.19 ± 7.82		16.68 ± 8.34		60.40 ± 9.82		43.77 ± 12.26		60.38 ± 16.26		50.05 ± 9.93	
>20	268	11.21 ± 9.28		12.19 ± 8.14		15.38 ± 9.15		59.14 ± 9.42		41.27 ± 9.71		61.32 ± 15.42		48.00 ± 7.89	
Faculty *															
Health	182	11.36 ± 8.38	0.641	12.76 ± 7.58	0.048	15.94 ± 8.70	0.239	58.52 ± 10.31	0.157	38.90 ± 9.78	0.463	60.76 ± 15.82	0.573	47.59 ± 7.99	0.663
Non-health	424	10.84 ± 8.47		11.61 ± 7.92		14.92 ± 8.58		60.55 ± 8.97		39.83 ± 9.59		61.40 ± 15.73		47.50 ± 6.94	
Marital status *															
Unmarried	597	11.51 ± 8.83	0.145	12.74 ± 7.91	0.540	16.14 ± 8.67	0.427	59.77 ± 9.60	0.439	42.21 ± 11.32	0.889	60.68 ± 15.92	0.048	49.18 ± 9.22	0.056
Married	9	8.67 ± 9.70		12.00 ± 10.82		13.78 ± 9.56		61.91 ± 12.12		41.20 ± 10.50		70.37 ± 11.85		43.74 ± 6.26	
Family support * No Yes	85 521	15.28 ± 8.95 11.56 ± 8.75	<0.001	16.52 ± 8.68 12.25 ± 7.66	<0.001	20.03 ± 8.60 15.66 ± 8.54	<0.001	55.69 ± 11.86 60.31 ± 9.30	0.001	41.24 ± 12.34 42.28 ± 11.20	0.496	53.86 ± 19.09 61.48 ± 15.24	0.001	46.75 ± 9.66 49.54 ± 8.99	0.019
History of COVID-19 diagnosed *															
Yes	83	13.86 ± 9.27	0.023	14.80 ± 9.65	0.055	17.47 ± 9.28	0.233	59.42 ± 11.26	0.870	41.62 ± 10.86	0.685	62.748 ± 15.80	0.153	49.75 ± 9.19	0.309
No	523	11.54 ± 8.75		12.40 ± 7.60		15.88 ± 8.59		59.86 ± 9.36		42.28 ± 11.38		60.51 ± 15.91		48.99 ± 9.21	
Family with COVID-19 diagnosed *															
Yes	251	12.76 ± 9.08	0.031	13.47 ± 8.28	0.098	17.43 ± 8.79	0.001	58.98 ± 10.91	0.095	41.19 ± 10.77	0.053	60.29 ± 16.86	0.686	49.21 ± 9.37	0.776
No	355	11.22 ± 8.64		12.21 ± 7.67		15.17 ± 8.52		60.38 ± 8.59		42.90 ± 11.63		61.20 ± 15.20		49.02 ± 9.09	
Comorbid disease *															
Yes	34	13.12 ± 8.39	0.241	15.18 ± 8.43	0.052	18.76 ± 9.09	0.096	56.42 ± 10.37	0.134	40.07 ± 8.95	0.270	57.35 ± 17.02	0.424	49.80 ± 9.92	0.489
No	572	11.79 ± 8.88		12.58 ± 7.90		15.95 ± 8.66		60.00 ± 9.56		42.32 ± 11.42		61.03 ± 15.82		49.06 ± 9.16	
Vaccination **															
Never	12	11.83 ± 10.11	0.283	12.00 ± 9.30	0.260	14.33 ± 9.60	0.081	59.22 ± 9.45	0.146	41.67 ± 12.81	<0.001	56.95 ± 18.41	0.138	47.03 ± 8.18	<0.001
1st	50	11.12 ± 8.72		11.56 ± 6.77		14.24 ± 8.75		60.93 ± 8.90		39.58 ± 9.60		58.83 ± 14.42		47.15 ± 8.31	
2nd	449	11.86 ± 8.75		12.69 ± 7.93		16.16 ± 8.57		59.32 ± 9.60		40.66 ± 10.07		60.32 ± 15.71		48.08 ± 7.98	
Booster	63	13.81 ± 9.28		14.38 ± 8.21		18.25 ± 8.74		62.08 ± 11.25		54.96 ± 12.81		64.41 ± 17.79		59.24 ± 11.21	
Headache * Yes No	127 479	14.54 ± 8.82 11.15 ± 8.73	<0.001	15.24 ± 7.71 12.06 ± 7.88	<0.001	18.98 ± 8.12 15.34 ± 8.70	<0.001	57.82 ± 10.49 60.32 ± 9.33	0.007	43.28 ± 11.57 41.90 ± 11.23	0.330	58.59 ± 17.28 61.41 ± 15.48	0.165	50.73 ± 9.94 48.67 ± 8.95	0.029
Fever * Yes no	66 540	15.36 ± 9.46 11.43 ± 8.68	0.001	16.00 ± 8.56 12.33 ± 7.78	0.001	19.55 ± 8.99 15.69 ± 8.58	0.002	56.71 ± 11.50 60.18 ± 9.32	0.032	44.83 ± 12.21 41.87 ± 11.16	0.042	57.96 ± 19.03 61.17 ± 15.46	0.507	50.29 ± 11.03 48.96 ± 8.95	0.225
Cough * Yes No	72 534	13.97 ± 10.27 11.58 ± 8.61	0.099	14.69 ± 9.08 12.46 ± 7.75	0.054	17.97 ± 9.47 15.85 ± 8.57	0.112	59.18 ± 11.41 59.88 ± 9.38	0.569	45.20 ± 13.11 41.79 ± 10.99	0.042	58.33 ± 19.02 61.16 ± 15.42	0.374	50.41 ± 10.97 48.93 ± 8.93	0.306
Itchy and Urticaria * Yes No	39 567	15.38 ± 8.76 11.65 ± 8.81	0.007	15.64 ± 8.82 12.55 ± 7.86	0.029	19.33 ± 7.60015.92 ± 8.73	0.006	58.88 ± 9.23 59.88 ± 9.67	0.465	43.91 ± 10.68 42.10 ± 11.34	0.318	57.48 ± 17.91 61.05 ± 15.77	0.220	50.62 ± 10.73 48.99 ± 9.06	0.719
Diarrhoea *Yes No	28 578	13.57 ± 10.80 11.76 ± 8.74	0.528	15.86 ± 8.73 12.53 ± 7.87	0.025	18.79 ± 9.83 15.97 ± 8.63	0.183	57.91 ± 11.05 59.87 ± 9.60	0.162	43.31 ± 12.49 42.13 ± 11.25	0.346	57.43 ± 11.64 60.97 ± 16.09	0.163	48.97 ± 9.94 49.11 ± 9.11	0.936
Myalgia * Yes No	42 564	18.00 ± 11.98 11.42 ± 8.41	0.001	16.57 ± 10.81 12.42 ± 7.62	0.034	20.57 ± 10.5215.78 ± 8.47	0.005	58.07 ± 12.84 59.92 ± 9.37	0.585	41.27 ± 14.43 42.27 ± 11.03	0.538	57.54 ± 18.57 61.03 ± 15.68	0.320	50.37 ± 10.46 49.05 ± 9.07	0.442
Sore throat * Yes No	49 557	17.74 ± 10.71 11.36 ± 8.50	<0.001	17.48 ± 8.66 12.28 ± 7.75	<0.001	21.26 ± 9.64 15.67 ± 8.49	<0.001	55.51 ± 13.89 60.13 ± 9.16	0.029	43.66 ± 16.12 42.07 ± 10.82	0.358	53.91 ± 20.56 61.43 ± 15.27	0.025	50.21 ± 12.23 48.98 ± 8.83	0.483
Anosmia * Yes No	14 592	15.29 ± 10.57 11.76 ± 8.79	0.208	13.86 ± 8.09 12.65 ± 7.94	0.597	18.43 ± 9.55 16.04 ± 8.69	0.537	55.35 ± 10.37 59.88 ± 9.64	0.055	40.49 ± 12.81 42.23 ± 11.27	0.511	58.33 ± 19.87 60.88 ± 15.81	0.919	48.61 ± 10.16 49.12 ± 9.13	0.770
Shortness of breath * Yes No	21 585	14.70 ± 7.23 11.75 ± 8.88	0.043	12.30 ± 4.91 12.69 ± 8.03	0.853	18.70 ± 8.16 16.01 ± 8.72	0.140	56.44 ± 10.08 59.89 ± 9.65	0.149	43.14 ± 10.49 42.16 ± 11.34	0.495	55.00 ± 18.01 61.00 ± 15.83	0.139	48.54 ± 11.75 49.12 ± 9.11	0.486

* Mann–Whitney, ** Kruskall–Wallis test.

**Table 7:** Linear regression models for depression, anxiety, and stress.

Variables	Depression			Anxiety			Stress		
	Regression Coefficient	Standard Error	*p*-Value	Regression Coefficient	Standard Error	*p*-Value	Regression Coefficient	Standard Error	*p*-Value
Intercept	38.622	4.586	<0.001	33.865	4.524	<0.001	36.299	4.632	<0.001
Gender	2.141	0.908	0.019 *	3.819	0.922	<0.001 *	3.968	0.992	<0.001 *
Age	−0.318	0.711	0.655	−0.885	0.727	0.224	−1.298	0.778	0.096
Family support	−3.303	1.087	0.002 *	−4009	1.116	<0.001 *	−3.749	1.200	<0.002 *
History of COVID-19 diagnosed	−2.278	1.016	0.025 *	−2.516	1.008	0.013 *	−0.907	1.161	0.435
Headache	−1.813	0.895	0.043 *	−1.648	0.892	0.065	−2.751	0.860	0.241
Myalgia	−4.280	1.410	0.003 *	−2.338	1.374	0.090	−3.988	1.444	0.006 *
Sore throat	−4.280	1.323	0.001 *	−4.359	1.302	0.001 *	−4.993	1.393	<0.001 *
Fever	−1.283	1.260	0.309	−1.729	1.391	0.182	−2.400	1.379	0.082
Cough	1.254	1.303	0.337	1.595	1.350	0.238	2.389	2.389	0.097

* Statistically significant.

**Table 8:** Linear regression models for physical health, psychological health, social relations, and environment.

Variables	Physical Health			Psychological Health			Social Relations			Environment		
	Regression Coefficient	Standard Error	*p*-Value	Regression Coefficient	Standard Error	*p*-Value	Regression Coefficient	Standard Error	*p*-Value	Regression Coefficient	Standard Error	*p*-Value
Intercept	42.556	5.426	<0.001	32.467	4.765	<0.001	11.314	9.683	<0.001	33.438	3.654	<0.001
Age	−1.874	0.923	0.043 *	−1.837	0.913	0.045 *	−0.330	1.573	0.834	−3.248	0.869	0.056
Family support	3.968	1.420	0.005 *	0.986	4.563	0.573	7.674	2.001	<0.001 *	2.897	1.117	0.010 *
Family with COVID-19 diagnosed	1.055	0.938	0.261	2.169	0.919	0.019 *	2.088	1.402	0.137	0.401	0.786	0.611
Vaccination status	0.754	0.870	0.386	5.454	0.875	<0.001 *	3.183	1.239	0.010 *	4.602	0.703	<0.001 *
Headache	1.952	0.999	0.051	−1.985	1.463	0.175	0.039	2.020	0.985	−2.023	1.046	0.054
Sore throat	3.955	1.634	0.016 *	1.365	2.249	0.544	7.379	2.378	0.002 *	0.892	1.780	0.617
Cough	−2.451	1.764	0.165	−2.939	1.404	0.037 *	−2.979	2.910	0.307	−1.999	1.669	0.231
Fever	2.799	1.317	0.545	−1.393	1.627	0.392	0.354	2.297	0.878	0.006	1.579	0.997

* Statistically significant.

**Table 9:** Correlation test to determine the relationship between MH and QoL.

Mental Health	Physical Health		Psychological Health		Social Relationship		Environmental Health	
	r	Sig 2	r	Sig 2	r	Sig 2	r	Sig 2
Depression	−0.393	<0.001 *	−0.161	<0.001 *	−0.400	<0.001 *	−0.040	0.325
Anxiety	−0.345	<0.001 *	−0.127	0.002 *	−0.242	<0.001 *	−0.013	0.756
Stress	−0.375	<0.001 *	−0.179	<0.001 *	−0.307	<0.001 *	−0.045	0.273

* Significant with and without Bonferroni correction.

## Data Availability

Data are contained within the article.
